# Ultrasensitive Detection of Testosterone Using Microring Resonator with Molecularly Imprinted Polymers

**DOI:** 10.3390/s151229877

**Published:** 2015-12-15

**Authors:** Yangqing Chen, Yong Liu, Xiaodan Shen, Zhimin Chang, Longhua Tang, Wen-Fei Dong, Mingyu Li, Jian-Jun He

**Affiliations:** 1 State Key Laboratory of Modern Optical Instrumentation, Zhejiang University, Hangzhou 310027, Zhejiang, China; chenyangqing@ioe-zju.org (Y.C.); liuyong@ioe-zju.org (Y.L.); 3110102785@zju.edu.cn (X.S.); lhtang@zju.edu.cn (L.T.); jjhe@zju.edu.cn (J.-J.H.); 2 CAS Key Lab of Bio-Medical Diagnostics, Suzhou Institute of Biomedical Engineering and Technology, Chinese Academy of Sciences, Suzhou 215163, Jiangsu, China; changzm@sibet.ac.cn (Z.C.); wenfeidong@sibet.ac.cn (W.-F.D.)

**Keywords:** Microring resonator, Molecularly imprinted polymers, Testosterone

## Abstract

We report ultrasensitive and highly selective detection of testosterone based on microring resonance sensor using molecularly imprinted polymers (MIP). A silicon-on-insulator (SOI) micoring resonator was modified by MIP films (MIPs) on a surface. The MIPs was synthesized by thermopolymerization using methacrylic acid as functional monomer and ethylene glycol dimethacrylate as crosslinking agent. The concentration of detected testosterone varies from 0.05 ng/mL to 10 ng/mL. The detection limit reaches 48.7 pg/mL. Ultrahigh sensitivity, good specificity and reproducibility have been demonstrated, indicating the great potential of making a cost effective and easy to operate lab-on-Chip and down scaling micro-fluidics devices in biosensing.

## 1. Introduction

Testosterone is a special steroid hormone that acts as the primary androgenic hormone. It is mainly secreted through the reproductive organs, playing key roles in human health. The testosterone levels depend on age, the typical value for male adults is 2.01~7.50 ng/mL [1]. Low testosterone levels can lead to serious problems like underdeveloped genitalia, abnormalities in skeletal and muscle development, and diminished masculinity [2]. A precise detection of testosterone levels is thus important to the study of medical and sports endocrinology [3]. Traditional blood sampling is limited as the testosterone levels are closely related to serum and plasma free concentrations [4]. A non-invasive measurement in saliva is preferred, as the salivary testosterone mainly exists in the free form rather than bounding to sex hormone binding globulin. This method is further beneficial as it eliminates the need of specialist sampling equipment and allows for large quantities of acquisition. Currently, numerous efforts have been devoted to detect testosterone, including enzyme-linked immunosorbent assay (ELISA) techniques [5], radioimmunoassay (RIA) [6,7], high performance liquid chromatography (HPLC) [8], gas chromatography-mass spectrometer (GC-MS) [9] and liquid chromatography-mass spectrometry (LC-MS) [10]. These time-consuming procedures require specialized personnel and expensive instrumentation, limiting them from been more extensively used. A more easy-to-use, economical and rapid method is thus expected.

Microring resonator represents one of the most vibrant research fields for optical sensor due to its supreme capability in label-free, rapid, ultra-sensitive and ultra-selective detection. The sensing light is coupled into the resonator with resonance condition, and constrained by waveguide surface with an evanescent field exponentially decaying into the surrounding medium [11,12]. The resonant wavelength is therefore affected by the refractive index of the solution in contact with the waveguide surface. Generally, microring resonators for sensing have focused on proof-of-principle investigations, such as improving performance of resonator [13], measuring in buffer of streptavidin-biotin interactions [14], detecting of proteins or virus [15]. Besides demonstrating the ability to monitor in real-time for the chemical modification and biological molecules binding of the sensor surface [16], the microring resonator also demonstrated to monitor multiplexed molecular binding simultaneously [17]. Previous reports concerning the recognition of biomolecules using microring resonator are mainly based on immunoassay techniques [18,19]. However, these recognition elements are time-consuming, unstable, typically possess high cost and only work under physiological conditions. Therefore, a robust and cost-effective recognition element is needed.

Molecular imprinting stands out as a promising method to create artificial receptors with molecular recognition sites [20]. The template molecule, functional monomer, crosslinking agent and initiating agent are aggregated into polymers followed by removing the templates to form recognition cavities [21]. The achieved MIPs are robust, stable and reproducible compared with natural material [22], representing an ideal alternative to biomolecules and having been widely used in biosensors [23,24] and other areas [25]. Usually, MIPs are combined with sensor by two typical methods. The first is that the pre-made MIP particles are immobilized on the surface of a sensor by physical capture [26]. This method forms a thick film and suffers from low sensitivity. The second utilizes in-situ self-assembly of MIPs directly in the surface of the sensor. Ultrathin film can be obtained and better sensitivity has been demonstrated compared to physical capture [27,28]. Different sensor structures have been applied in the second approach [24,29].

In this study, MIPs were prepared in the surface of microring resonator to detect testosterone by thermal polymerization. The template molecule and functional monomer were firstly treated with pre-polymerization to form self-assembled monolayer (SAM). The initiating agent was then covalently coupled to the carboxyl-terminated SAM, followed by MIPs directly immobilized in surface of the sensor during polymerization. The fabricated MIPs sensor was used to detect different concentrations of testosterone. The selectivity of the MIPs sensor was also evaluated by detecting microcystin-LR. Subsequently, the sensor was regenerated by an acetic acid-ethanol solution. Ultrahigh sensitivity, good specificity and reproducibility have been demonstrated, promising for real-time and low-cost salivary testosterone detection.

## 2. Experimental Section

### 2.1. Reagents

Methacrylic acid (MAA), Ethylene glycol dimethacrylate (EGDMA), acetonitrile and acetic acid were purchased from Sigma-Aldrich (Schnelldorf, Germany). Testosterone and 2,2’-Azobisiobutyronitrile (AIBN) were purchased from Sinopharm Chemical Reagent Co. Ltd (Shanghai, China). All other reagents were purchased from Sinopharm Chemical Reagents Co. Ltd (Shanghai, China).

### 2.2. Instrumentation and Microring Sensor

**Figure 1:**
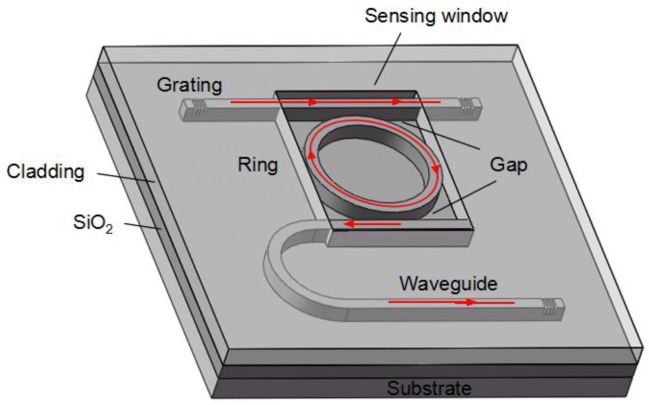
Schematic of the microring resonator sensor.

**Figure 2:**
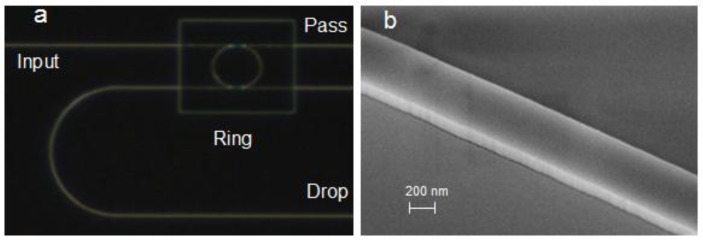
(**a**) Optical microscope image of the microring resonator; (**b**) SEM image of the waveguide.

The instrumentation to measure transmission spectrum of microring resonator contains a tunable light source (Agilent 81600B) coupled into input waveguide and a power meter (Agilent 81635A) to collect the output light. Sensor chips employing grating couplers were manufactured on silicon-on-insulator (SOI) wafers with a 220 nm-thick top silicon layer and 2 µm-thick buried oxide layer, as shown in Figure 1. Figure 2a shows the optical microscope image of the microring resonator sensor and Figure 2b shows the scanning electron microscopy (SEM) image of the waveguide. The radius of ring is 32 µm and the length of directional coupler is 8 µm. In addition, the width of waveguide and gap are 500 nm. All the strip waveguides are deep etched with 220 nm except grating couplers are shallow etched with 70 nm. The whole sensor is coated by SiO_2_ with 2 µm-thick upper cladding layer while the microring resonator is exposed to the analyzed sample by removing the upper cladding layer in the sensing window.

### 2.3. MIPs Synthesis

**Figure 3:**
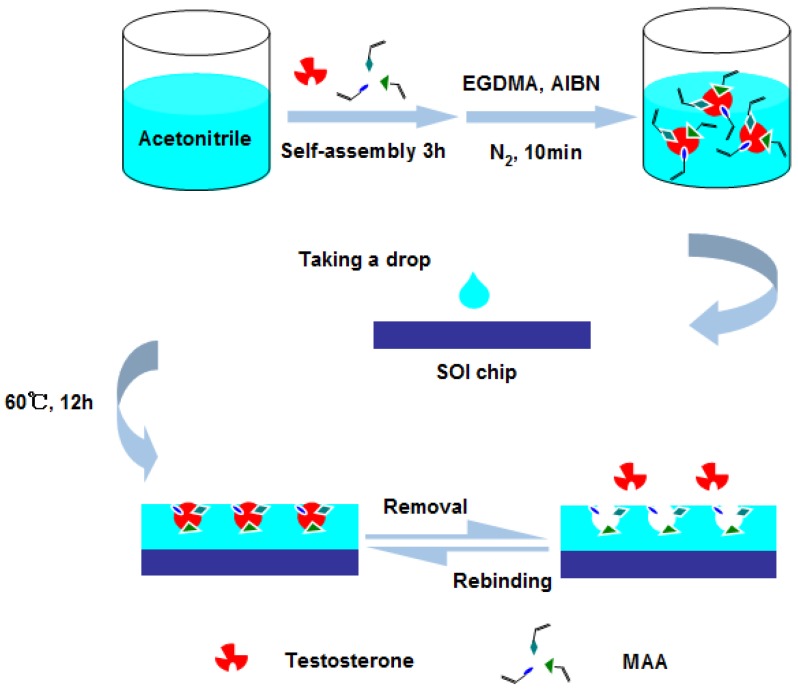
Schematic for preparation molecularly imprinted polymers on the surface of chip.

At first, the sensor chip was cleaned by acetone, ethanol and deionized (DI) water for 5 min individually, then dried using nitrogen. As illustrated by the schematic in Figure 3, for the preparation of self-assembly, 5 mg of testosterone and 400 µL of functional monomer MAA were added into 2 mL of acetronitrile and placed at 25 °C for 3 h. Subsequently, 0.5 mL of crosslinking agent EGDMA and 9 mg of initiating agent AIBN were appended to the solution. After being treated with nitrogen gas for 10 min, the reaction solution was cast onto the surface of sensor chip. The sensor chip was placed in a hot-air oven at 60 °C for 12 h for polymerization. After the polymerization, the sensor chip was washed by an acetic acid-ethanol solution (volume ratio is 1:1) to remove potential residual organics and the template molecule. At last, the sensor chip was rinsed by DI water and dried by nitrogen. Furthermore, non-imprinted polymer films (NIPs) were synthesized without using the testosterone as template molecule, and were used to evaluate the specificity of the sensor.

### 2.4. Testosterone Detection

The solution of testosterone is made by serial dilution in DI water. Before each measurement, the MIPs sensor was taken out and stabilized for several minutes. Then, the sensor chip is exposed to testosterone solution with different concentrations. The transmission spectrum for each solution is measured. The sensor chip is washed by an acetic acid-ethanol solution (volume ratio is 1:1), cleaned by DI water and dried by nitrogen after each measurement.

## 3. Results and Discussion

### 3.1. Quantitative Detection

**Figure 4:**
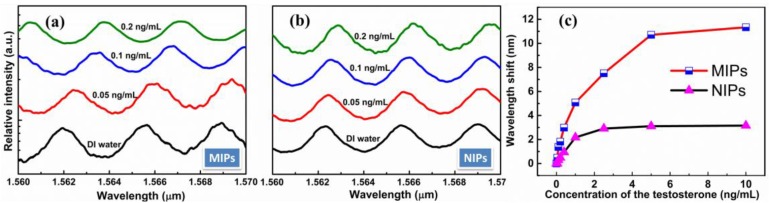
(**a**) Transmission spectra of molecularly imprinted polymer films (MIPs) when detecting testosterone; (**b**) Transmission spectra of non-imprinted polymer films (NIPs) when detecting testosterone; (**c**) Wavelength shift of MIPs and NIPs film coated chips, for the detection of different concentration of testosterone.

The adsorption properties of sensor were characterized by the shift of resonant wavelength. The affinity binding of testosterone molecules was detected by a series of testosterone samples with the concentrations ranging from 0.05 to 10 ng/mL. The transmission spectra and the wavelength shift of the sensor chips coated by the MIPs and NIPs respectively, in the different concentrations of testosterone were showed in Figure 4. From Figure 4a,b, a remarkable resonant wavelength shift of sensor coated by the MIPs can be clearly observed when changing the concentration of testosterone from 0.05 ng/mL to 0.2 ng/mL, but the resonant wavelength of the sensor coated by the NIPs is not significantly shift. Furthermore, the sensitivity of sensor is S = 4.803 nm/ng·ml^−1^, which obtained by fitting as shown in Figure 4c. We adopt the traditional method of using 3 times standard deviations σ as a measure of the sensor resolution [30] R = 3σ = 0.234 nm, while σ = 0.078 nm depends on the total system noise and the spectral resolution. Furthermore, the detection limit L = R/S = 48.7 pg/mL were obtained. This result suggested that the microring resonator coated by MIPs can adsorb the template molecules and ultrahigh sensitivity.

### 3.2. Specific Recognition

**Figure 5:**
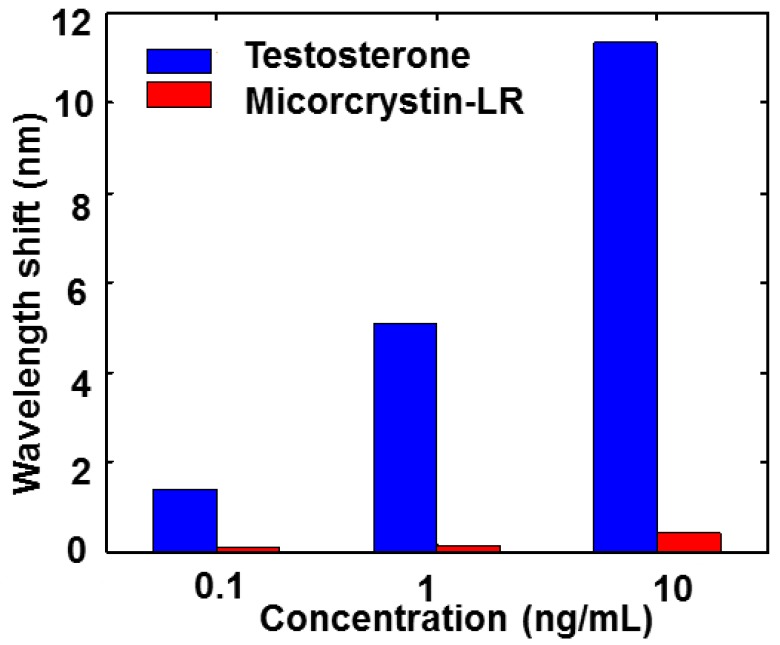
Wavelength shift of MIPs film coated chips, for the detection of different concentration of testosterone and microcystin-LR.

The microcystin-LR was used to evaluate the specificity of the MIPs coated chip response. As shown in Figure 5, results indicate that the sensor was only sensitive to testosterone, but not to microcystin-LR molecular. The results strongly demonstrated that the sensor had a good specific recognition for testosterone.

### 3.3. Reproducibility

**Figure 6:**
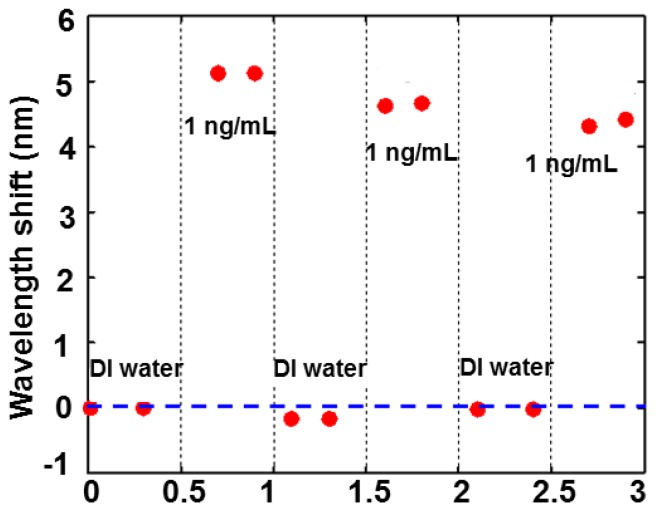
Resonant wavelength shift of the sensor for 1 ng/mL testosterone after each regeneration.

To evaluate the reproducibility of the MIPs, the microring resonator chip was regenerated by rinsing in an acetic acid-ethanol solution (volume ratio is 1:1) and DI water, dried by nitrogen after each measurement. The transmission spectrum was re-measured in the same condition and the comparison of resonant wavelength shift was shown in Figure 6. The fabrication process of MIPs indicated that the MIPs can be regenerated many times. However, the response of the sensor has a drift for 1 ng/mL testosterone in the test of the reproducibility as shown in Figure 6 because the MIPs were damaged a little when testing. This limits the maximal number of the sensor regenerations.

## 4. Conclusions

The reported biosensor has achieved a low detection limit of 48.7 pg/mL based on a combination of microring resonator and self-assembled MIPs. The MIPs can be easily grown on the sensor surface with simple procedures. Ultrahigh sensitivity, good specificity and reproducibility of the sensor have been demonstrated. This robust, stable and reproducible assay system is strategically important for salivary testosterone detection in near real-time clinical diagnostics.
